# Migrating Ovaries: Early Life Influences on Later Gonadal Function

**DOI:** 10.1371/journal.pmed.0040190

**Published:** 2007-05-15

**Authors:** Peter D Gluckman, Alan S Beedle

## Abstract

The authors discuss a new study of Bangladeshi migrants to the UK, which found that adult women who had migrated before the age of 8 years had greater luteal phase progesterone secretion than those who had migrated after that age.

Progress in the biomedical and clinical sciences has relied heavily on experimental animal research. However, the impact of other comparative disciplines such as evolutionary biology, and in particular its subdisciplines of life history biology and ecological developmental biology, on human medicine has been limited. Despite its heritage and contributions, evolutionary biology has rarely been applied to understanding human development, particularly from a medical rather than an anthropological perspective.

**Linked Research Article**

This Perspective discusses the following new study published in *PLoS Medicine:*

Núñez de la Mora A, Chatterton RT, Choudhury OA, Napolitano DA, Bentley GR (2007) Childhood conditions influence adult progesterone levels. PLoS Med 4(5): e167. doi:10.1371/journal.pmed.0040167

This migrant study involved first- and second-generation Bangladeshi women living in the UK, women still resident in Bangladesh, and women of European descent in the UK. The study found that women who spent their childhood in conditions of low energy expenditure, stable energy intake, good sanitation, low immune challenges, and good health care had higher levels of progesterone and an earlier maturation than women who developed in less optimal conditions.

Recently this gap in applying evolutionary biology to medicine has started to close, with a growing recognition that this discipline is critical to understanding the human condition and the risks of health and disease. Randolph Nesse and colleagues have proposed that evolutionary biology and “evolutionary medicine” should be a core discipline of medical training [1]. For example, evolutionary biology helps in understanding “lifestyle-associated” diseases such as type 2 diabetes mellitus [2]. Developmental plasticity, through the induction of a range of phenotypes from a single genotype in response to developmental cues, has a crucial role in determining the risk of such diseases [2]. The impetus for understanding the role of developmental biology in disease causation was the research by David Barker and colleagues who recognised the relationship between the conditions of the fetus as reflected in birth size and the later risk of disease [3]. This research in turn built on an early body of work, albeit somewhat less clear in its interpretation, relating the conditions of early life to later mortality (see [4] for review). A strong body of experimental studies has now linked the perinatal state to later metabolic function (reviewed in [5]). Most recently, the role of epigenetic processes (environmentally sensitive modifications of the genome that allow stable transmission of gene expression states without alteration of the DNA sequence) in linking these early life events to later consequences, potentially crossing generations [6], has become apparent [7].

## Life History Trade-Offs and Reproductive Strategy

Life history theory is a framework developed to explain the very different strategies of growth, maturation, and reproduction used by different species across different environmental niches. The framework also attempts to explain the variation in reproductive and other strategies that individuals in a species may adopt, particularly when in different energetic states. Simply put, it posits that the organism must trade off its limited energetic capacity against different key components of its life course strategy so as to maximise the probability of gene flow to the next generation.

Selection acts on the mechanisms underlying this trade-off capacity. Important trade-offs affect patterns of reproductive and mating strategy as well as patterns of growth and relative investment in repair, and thus lifespan. There is an extensive theoretical basis for this approach (see, for example, [8]) supported by much empirical data, particularly in insects and amphibia.

In human biology, life history theory is applied to explain biodemographical observations such as patterns of reproduction and ageing, often obtained from studies in modern hunter-gatherer communities. In human females, trade-offs affecting reproductive strategy could emerge as differences in ages of menarche or menopause, in fecundity, or in inter-birth interval. For example, a recent study showed a trade-off between the age of menarche and mortality risk across a broad sample of hunter-gatherer societies [9]. Clearly, for most such trade-offs to operate they must be defined early in development. There is an emergent theory addressing how early life cues such as nutrition can influence phenotypic development and the expression of traits such as elements of reproductive performance [10], operating through the processes of changed epigenetic expression [11].

Evidence for developmental effects of environment on mammalian reproductive strategy is increasing. For example, in sheep it is well documented that seasonality of birth (and thus, indirectly, nutrition) determines the timing of puberty [12]. In humans, it has been suspected for some time that there is a relationship between nutritional status in late childhood and the age of menarche. The now discredited Frisch hypothesis tried to tie menarche to a specific level of body fat, but evidence across populations links better nutritional status in late childhood to early menarche [13,14]. At the mechanistic level this link may well involve the interplay between the adipokine leptin and the neuroendocrine regulation of gonadotropin secretion [15]. Conversely, there is also evidence, from both experimental and epidemiological studies, that fetal undernutrition accelerates the age of menarche, particularly if later childhood nutrition is good [13,16,17]. This dichotomy can be seen within the same cohort, and in life history terms can be explained as two different maturational signals—that before birth signalling a later threatening world in which gene flow is protected by early reproductive competence, and a postnatal override that samples nutritional status as an indication of whether current energy provision is sufficient to sustain pregnancy.

This override or a similar mechanism also operates beyond puberty, as shown by the well-established relationship between energy balance and ovarian function in adult women [18]. The set point of this relationship can be modified by prenatal energetic constraints, since the ovarian function of women born thinner, and thus with predictive adaptations towards success in a harsher environment, is more sensitive to physical workload than that of women of greater fatness at birth [19].

## Further Evidence for Developmental Effects on Ovarian Function

In a new paper published in *PLoS Medicine*, Alejandra Núñez-de la Mora et al. [20] take the evidence for early life effects on later ovarian function a step further. In a study of Bangladeshi migrants to the United Kingdom they evaluated adult luteal function (as indicated by salivary progesterone levels) and age at menarche. They compared groups who had migrated at different ages, had migrated in a previous generation, or had not migrated. While such studies are inevitably at risk of confounding factors such as current nutrition or workload, the authors made a credible attempt to control for these variables. They found a substantial effect: mature women who had migrated before the age of 8 years had higher luteal phase progesterone secretion than did those who migrated after that age. In this respect, the former group behaved like second-generation migrants and Europeans rather than women who had stayed in Bangladesh. The earlier the women had migrated as children, the earlier the age of menarche—supporting the general conclusion that good childhood conditions accelerate menarche.

This finding adds considerable weight to the utility of applying life history concepts to the study of human reproduction. Such perspectives may be important in understanding the conflict between the sociological drive to later first pregnancy and the declining fertility of older women, particularly those attempting their first pregnancy, who then must face the increased use of assisted reproductive technologies. Whether the conditions of early life are intertwined with the pattern of later reproductive decline merits further research.

## Clinical Implications

In the final part of their paper the authors speculate that the increased adult levels of progesterone experienced by younger migrants may play a role in the changing incidence of breast cancer. This is a rather broad extrapolation from their data, and there are many other possible explanations for the epidemiological observations. Yet there is other evidence associating early life events with the changing risk of breast cancer—larger babies have a greater risk of premenopausal breast cancer, for which several alternative mechanisms, including increased levels of intrauterine growth factors, have been proposed [21]. Thus, although there may not be a direct causal link between progesterone levels and cancer incidence, the authors are indeed correct to point out that influences in fetal life, infancy, and childhood can affect not only later ovarian function but potentially the risk of reproductive malignancies.

This paper reminds us of the broader importance of considering early life effects in human medicine. The “developmental origins” model originally focused on the effect of prenatal nutrition on later risks of disease [3] and was later extended to show the compounding effects of both poor fetal growth and excessive infant growth [22]. The evidence is mounting that although the model is based on a disease outcome, it reflects normative mechanisms that have evolved to adjust the individual's development to match the anticipated environment [2]. That paradigm is now being extended to include other components of phenotypic variation, including influences on gonadal and other endocrine function. The paper by Núñez-de la Mora et al. [20] underscores that variation in adult ovarian function, which might manifest as altered fertility and fecundity or perhaps as altered risk of hormone-associated disease, has a developmental component. Such fundamental biological perspectives have much to offer clinical medicine.
