# Correction: Migrating Ovaries: Early Life Influences on Later Gonadal Function

**DOI:** 10.1371/journal.pmed.0040226

**Published:** 2007-06-26

**Authors:** Peter D Gluckman, Alan S Beedle

In *PLoS Medicine*, volume 4, issue 5, doi:10.1371/journal.pmed.0040190:

Reference 22 was truncated. The full reference should be:

22. Barker DJ, Osmond C, Forsen TJ, Kajantie E, Eriksson JG (2005) Trajectories of growth among children who have coronary events as adults. N Engl J Med 353: 1802–1809.

